# The status of IT service management in health care - ITIL^® ^in selected European countries

**DOI:** 10.1186/1472-6947-11-76

**Published:** 2011-12-21

**Authors:** Alexander Hoerbst, Werner O Hackl, Roland Blomer, Elske Ammenwerth

**Affiliations:** 1 Research Division eHealth and Telemedicine, UMIT - University for Health Sciences, Medical Informatics and Technology, Eduard-Wallnoefer-Zentrum 1, Hall in Tirol, Austria; 2 Institute for Health Information Systems, UMIT - University for Health Sciences, Medical Informatics and Technology, Eduard-Wallnoefer-Zentrum 1, Hall in Tirol, Austria

## Abstract

**Background:**

Due to the strained financial situation in the healthcare sector, hospitals and other healthcare providers are facing an increasing pressure to improve their efficiency and to reduce costs. These trends challenge health care organizations to introduce innovative information technology (IT) based supportive processes. To guarantee that IT supports the clinical processes perfectly, IT must be managed proactively. However, until now, there is only very few research on IT service management especially on ITIL^® ^implementations in the health care context.

**Methods:**

The current study aims at exploring knowledge about and acceptance of IT service management (especially ITIL^®^) in hospitals in Austria and its neighboring regions Bavaria (Germany), Slovakia, South Tyrol (Italy) and Switzerland. Therefore highly standardized interviews with the respective head of information technology (CIO, IT manager) were conducted for selected hospitals from the different regions. In total 75 hospitals were interviewed. Data gathered was analyzed using descriptive statistics and where necessary methods of qualitative content analysis.

**Results:**

In most regions, two-thirds or more of the participating IT managers claim to be familiar with the concepts of IT service management and of ITIL^®^. IT managers expect from ITIL^® ^mostly better IT services, followed by an increased productivity and a reduction of IT cost. But only five hospitals said to have implemented at least parts of ITIL^®^, and eight hospitals stated to be planning to do this in the next two years. When it comes to ITIL^®^, Switzerland and Bavaria seem to be ahead of the other countries. There, the highest levels of knowledge, the highest number of implementations or plans of an implementation as well as the highest number of ITIL^® ^certified staff members were observed.

**Conclusion:**

The results collected through this study indicate that the idea of IT services and IT service management is still not widely recognized in hospitals in the countries and regions of the study. It is also indicated that hospitals need further assistance in order to be able to successfully implement ITIL^®^. Overall, research on IT service management and ITIL^® ^in health care is rare.

## Background

Due to the strained financial situation in the healthcare sector, hospitals and other healthcare providers are facing an increasing pressure to improve their efficiency and reduce costs while maintaining or even increasing the variety and quality of their medical services. Besides that, general medical progress leads to a constant increase in knowledge and a higher specialization of medical professionals. Further we can observe a constant sophistication of medicine with introduction of new technologies for diagnosis, therapy and rehabilitation. We can also observe an increased mobility of patients, a continuous shift in the age distribution in western countries, and an increase of empowerment and self-determination of patients [1].

All these trends lead to a rising demand for innovative information technology (IT) based service processes in health care organizations. IT tools are used to provide the correct and relevant information quickly and comprehensively at the right place, the right time and for the right people at reasonable costs, and they help to reach a high level of quality and user satisfaction [2,3]. Health care organizations typically use various computer-based information systems to support and even enable their medical and organizational processes. To guarantee that IT supports the business, IT must proactively manage its own processes which accompany and support the respective business processes. A process based proactive management of IT services which covers the whole service management life cycle is called IT service management (ITSM). However, until now, there is only very few research on ITSM in general and especially on ITIL^® ^implementations in the health care context (for example [4]). Besides of ITIL^® ^there are a number of other approaches to implement IT service management within an organization such as eTOM (enhanced Telecom Operations Map) [5], MOF (Microsoft Operations Framework)[6], ITPM (IT Process Model) [7], HP-ITSM (Hewlett Packard IT Service Management) [8] or IT Service CMM (Capability Maturity Model) [9]. But ITIL^® ^is one of the most advanced, generic and widely used frameworks for ITSM. The following paragraph briefly introduces ITSM and the ITIL^® ^approach.

### IT service management

Information technology service management (ITSM) in general is concerned with the management of IT services through the use and coordination of people, workflows and information technology [10]. These three components are the heart of business processes. Health care can be considered as a special business. Business processes are commonly classified as: strategic business processes, core business processes, and supportive business processes. IT processes belong to the class of supportive business processes which deliver outputs in terms of services needed by the business. ITSM supports the implementation and management of high quality IT-Services. ITSM is performed by IT Service Providers through processes [11]. It mainly differs from traditional IT management approaches as it is no longer technology-centered but looks at IT and its services from a business perspective. This becomes also manifested as ITSM is in close relationship with other approaches for quality such as Total Quality Management or Six Sigma.

### IT Infrastructure Library (ITIL^®^)

ITIL^® ^provides a framework for the implementation of the ITSM approach in an organization and is based on a number of best practices. ITIL^® ^aims at providing high quality IT services taking both the business and customer perspective into account. ITIL^® ^was developed in the UK on behalf of the Central Communications and Telecommunications Agency. Since its dawn in the late 1980s, ITIL^® ^has evolved and was constantly improved. The first version of ITIL^® ^has influenced and inspired several other approaches to ITSM. ITIL^® ^is currently available in Version 3 [12]. The contents of Version 3 are aligned according to the lifecycle of a service and are therefore structured in the following five core domains:

• *Service Strategy*: deals with the concept and strategy with regard to IT services during their lifecycle; apart from service definition and specification also service logistics and financial aspects are described from a strategic business perspective.

• *Service Design*: is focused towards the definition of services and service management processes. It includes guidelines and principles to operationalize strategic goals defined by the service strategy including service logistics or financial aspects.

• *Service Transition*: provides necessary processes and methodologies to transform service strategy requirements and service design aspects into operational services including means to reduce errors and failures by implementing release, risk and program management.

• *Service Operation*: describes ways to efficiently and effectively deliver services for the daily operation. Topics covered are service support, service stability or the constant delivery of an agreed service level.

• *Continual Service Improvement*: aims at constantly maintaining and improving service quality and therefore customer satisfaction by influencing the service design, service introduction and service operation phases. It contains and combines quality management, change management and process improvement by linking these methodologies to the respective phases in the lifecycle of a service.

Overall, the current version of ITIL^® ^describes 26 processes, reaching from the strategic alignment to the continual improvement of IT services. Apart from the processes, Version 3 also contains a set of functions: Service Desk, Application Management, IT Operations Management, IT Operations Control, IT Facility Management, and Technical Management.

ITIL^® ^certification is granted to individuals, but not to organizations. The ICMB (ITIL^® ^Certification Management Board) defines and manages a hierarchy of certification levels for individuals.

#### ISO/IEC 20000 series

While ITIL^® ^allows certification only on an individual level, ISO/IEC 20000 allows organizations to certify their service management system. ISO/IEC 20000 is a series of standards that can be implemented by organizations that provide IT services to internal and external customers. The series consists of five parts. Part one [13] specifies requirements with regard to the service management system whereas part two [14] contains guidance information based on an industry consensus on service improvement and auditing against part one. ISO/IEC 20000 is based on an integrated process approach to IT service management and adopts the processes of the ITIL^® ^framework within a process model which is itself part of the definition of a quality management system. Talking about the relation of ITIL^® ^and the ISO/IEC 20000, ITIL^® ^provides organizations with guidelines of how to align IT services and ITSM with the business whereas the ISO/IEC standard aims at benchmarking the quality of an organization's IT service management.

### Objectives

In many business domains, ITIL^® ^has been adopted as the de-facto standard for IT service management. However, it seems unclear to what extent the idea of IT service management and ITIL^® ^has been recognized and adopted in health care.

The current study tries to gain more insight on how far IT service management and especially ITIL^® ^are known, are being accepted and have penetrated health care organizations in Austria and in the neighboring regions.

### Investigated Regions

The study at hand was conducted in Austria and its neighboring regions Bavaria (Germany), Slovakia, South Tyrol (Italy) and Switzerland. The countries and regions investigated are situated in Central Europe and are inhabited by approximately 34 million people. The following paragraphs describe structural parameters concerning the health systems of the five countries or regions studied.

#### Austria

The Republic of Austria, member of the European Union since 1995, had a population of over 8.3 million inhabitants and a total expenditure on health (THE) of 10.1% of its GDP in 2008 [15]. Austria had 269 hospitals offering 64556 beds (775 beds per 100000 inhabitants) in 2007. The total number of hospital stays was 2.7 million; the average length of a stay was 5.64 days [16]. Austria operates a DRG system to reimburse the hospitals. A national, mandatory social insurance system (compulsory contributions) grants free access (with humble patients' contributions) to public healthcare.

#### Bavaria

The Free State of Bavaria is the second largest (with respect to population) of the 16 German states with a total population of approximately 12.5 million inhabitants. Germany is one of the founding members of the European Union. In 2008 there were 373 hospitals with a capacity of 75374 beds (603 per 100000 inhabitants) in Bavaria. The total number of hospitalizations in 2008 was 2.7 million with an average length of stay of 8.0 days [17]. Germany (including Bavaria) also has a compulsory public health insurance system with almost free access to intramural health care and has implemented a DRG system to finance the hospitals. THE was 10.4% in 2008 in Germany [15].

#### Slovakia

The Slovak Republic, established in 1993 is European Union member since 2004 and has a population of 5.4 million inhabitants. THE as percent of GDP was 7.8 in 2008 [15]. The Slovakian health care services are also free of charge based on a mandatory health insurance obligation. In 2008, 179 facilities for institutional care offered 35678 hospital beds (660 per 100000) for the Slovakian citizens. In the same year 1012400 hospitalized patients were counted; the average length of stay was 8.5 days [18].

#### South Tyrol

The autonomous Province of Bolzano-Bozen (also referred to as South Tyrol) is the most northern province of Italy. More than two-thirds of the about 500000 inhabitants are German-speaking [19]. The primary health care is maintained by eight (seven public plus one private) hospitals. Due to a bilateral agreement the University Hospitals in Innsbruck (Austria) can also be frequented by citizens from South Tyrol. In 2007 a reform of the health system centralized the administration (including the IT departments) for the public hospitals [20]. The health system in South Tyrol had a capacity of 2143 beds (435 per 100000 inhabitants) in 2008. In the same year 98889 stays with an average duration of 6.8 days were counted [21].

#### Switzerland

The Swiss Confederation is a federal republic with four official languages and has a population of approximately 7.7 million inhabitants. Switzerland is member of the European Free Trade Association (EFTA), but not a member of the European Union. The Swiss hospitals are regulated by cantonal legislation and therefore quite heterogeneous. As an inhabitant of Switzerland everyone is obligated to choose one of about 90 available health insurances which then cover the expenditures for healthcare. The rates are dependent on an individual selectable participation, age and health status [22]. In 2008 Switzerland spent in total 10.5 percent of its GDP for the health system [15].

In 2008, 318 hospitals were providing 41352 beds (537 per 100000 inhabitants). The average duration of the more than 1.2 million stays was 10.7 days in the respective year [23].

## Methods

The subsequent section describes the method used for the current study including information on the general research questions/problem, the selection and size of the sample and the collection as well as the analysis of data.

### Research problem and questions

The research problem was defined according to chapter 2. Objectives:

• It is unknown to what extend IT service management respectively ITIL^® ^is known to, accepted by and implemented by health care organizations in Austria and in its neighboring regions.

The research questions investigating the research problem were defined as follows:

1. How is the IT organized in the different hospitals?

2. How advanced is the knowledge of and the attitude about IT service management in general and especially about ITIL^® ^among IT managers in the different hospitals?

3. How advanced are the different hospitals with regard to the implementation of ITIL^®^? What are the reasons that hinder the implementation of ITIL^®^?

4. Which differences between hospitals of the different regions/countries can be observed?

### Study design

The specific design for the current study is based on a survey research method [24], making use of standardized questionnaires and predominantly personal as well as telephone interviews. The study design was discussed and verified with the responsible persons of our university's ethics committee. A formal approval of the design was not considered necessary by the responsible. The data analysis is based upon descriptive statistics. The survey method was selected as it's especially suitable to gain better understanding of a problem and its reasons by quantifying certain aspects of it [25]. Although trying to quantify certain phenomena the study is primarily intended to explore the given problem context in order to enable further detailed and hypothesis-based research. The following subsections describe the chosen design in detail.

### Sample selection and sample size

In order to gather a meaningful sample from the different countries/regions, the structural distribution of existing hospitals was analyzed according to basic parameters including the number of beds, type of hospital, type of owner and the area of responsibility. This information - mainly collected from official statistics and hospital listings - was correlated with the federal structure of the respective countries/regions.

From the resulting list the largest hospitals with regard to beds and responsibility in each canton/region/province were picked to be included in the study, as in the case of ITIL^® ^a critical size is necessary in order to be reasonably implemented. Subsequently details are given for each country/region with regard to the sample selection and size.

#### Slovakia

33 organizations from 8 different regions in Slovakia were selected randomly to participate. The relative number of hospitals selected is the same for each region in Slovakia depending on the total number of hospitals for that region. In Slovakia there are nine university hospitals which were all included regardless of the region they belong to.

#### Switzerland

In Switzerland an average of three hospitals was picked from each of the 26 cantons. It was again aimed at picking a number of hospitals for each canton that is relatively equal to the number of hospitals selected in other cantons. Similar to Slovakia, all five university hospitals were included in the sample.

#### Austria

In Austria five hospitals were selected from each federal state yielding a total of 45 hospitals which were included in the sample. All university hospitals are included in this sample.

#### Bavaria

In Bavaria 35 hospitals were selected in total, five each from its seven administrative regions.

#### South Tyrol

As the number of existing hospitals is comparably low, all - one private and seven public - hospitals located in the province of South Tyrol were added to the sample. Due to several administrative reforms, the latest in 2007, several public hospitals are grouped together with regard to their administration (including their IT). Therefore the total number of independent hospitals for South Tyrol is five.

### Data collection

In order to answer the research questions, highly standardized interviews with the respective head of information technology (CIO, IT manager) - indicated by the respective organizations - were conducted for the selected hospitals. The interview questions were mostly closed questions with a given set of possible answers. All interviews were conducted in the second and third quarter of 2008 either personally, by telephone, or online. The interviews were based on previously designed and tested interview guidelines. These interview questions are grouped in three main sections containing four open and 58 closed questions:

• General questions with regard to the healthcare organization (nine questions). This group of questions includes information such as type of organization, number of beds or hospital owner.

• Questions with regard to the use of IT in the healthcare organization to determine the organizational and technical maturity of the organizations (33 questions). Topics covered by this section are organization of IT including financing, current and planned IT systems, technical equipment and customer- and user relationship management.

• Questions with regard to the current implementation of ITSM/ITIL^® ^in the organization and the potential interest and willingness to implement ITSM/ITIL^® ^(20 questions). Questions included cover awareness and use of ITSM in general, awareness, use or intended use of ITIL^®^.

The guidelines were translated to the official language of the country or region concerned. In Switzerland, due to the variety of languages spoken, English guidelines were used for questioning.

The interviews were carried out by one single interviewer for each country/region. To assure comparable conditions for the interviews, guidelines for the different interviewers were developed. These guidelines described rules as how to pose questions and how to document the answers given. Furthermore, all interviewers were trained together in pretest interviews.

The time needed for completing an interview showed great variability ranging from 6 to 50 minutes depending on the total number as well as the level of detail of answers. In total, 75 organizations provided valid data (return rate: 47.5%; see Table 1).

**Table 1:** Overview of hospitals contacted and response rate in the different countries/regions

Country/Region	Hospitals contacted	Hospitals participated
Slovakia	33	23 (69.7%)

Switzerland	65	30 (46%)

Austria	45	16 (35.5%)

Bavaria	35	13 (37.1%)

South Tyrol	5	5 (100%)

Overall	183	87 (47.5%)

### Data analysis

The collected data from the interviews was basically analyzed by using SPSS Statistics Version 17 and Microsoft Excel 2003.

For the closed questions all predefined answers were coded and actual answers given by interviewees assigned. This was done by each interviewer for his/her country.

To analyze the answers to open questions or the answers of closed questions where interviewees could provide additional explanations, techniques of content analysis according to Mayring [26] were applied. Categories were inductively derived from the answers by abstracting and generalizing them. Due to the limited number and/or homogeneity of answers provided for open questions by the interviewees the construction of the categories for each question required only one revision cycle - having processed about 40% of the interviews at this time - before being consistent. Afterwards categories were coded and answers from the interviews assigned.

Subsequently all results from the different countries were merged before the final analysis of the results. The final analysis is based on descriptive statistics indicating the relative or absolute frequency of answers with regard to country and question. Where applicable also the number of non respondents is indicated and added to the tables. Some questions were dependent on the answers of other questions therefore the sample size sometimes varies and hence is indicated for each table.

## Results

In the subsequent chapters the results from the study are described. The presentation of the results follows the structure of the interview guidelines.

### Basic organizational data about hospitals

Table 2 gives an overview of the types of the participating 75 hospitals. University Hospitals, Specialized Hospitals, and General Hospitals were mostly evenly distributed in the overall sample.

**Table 2:** Types of hospitals participating in the study.

	Slovakia		Switzerland		Austria		Bavaria		South Tyrol		TOTAL	
	
	n	%	n	%	n	%	n	%	n	%	n	%
University Hospital	**11**	**48%**	1	6%	2	13%	2	15%	0	0%	16	21%

Specialized Hospital	3	13%	1	6%	4	25%	**8**	**62%**	**3**	**60%**	**19**	**25%**

General Hospital	5	22%	**8**	**44%**	4	25%	2	15%	1	20%	20	27%

Other	4	17%	**8**	**44%**	**6**	**38%**	1	8%	1	20%	20	27%

TOTAL	23	100%	18	100%	16	100%	13	100%	5	100%	75	100%

The maximum in each column is highlighted.

Table 3 provides an overview of the ownership of the participating hospitals. The majority of the participating hospitals are in public ownership (Total: 59%, n = 44).

**Table 3:** Ownership of hospitals involved in the study.

	Slovakia		Switzerland		Austria		Bavaria		South Tyrol		TOTAL	
	
	n	%	n	%	n	%	n	%	n	%	n	%
Public	**13**	**57%**	7	39%	**9**	**56%**	**11**	**85%**	**4**	**80%**	**44**	**59%**

Private	2	9%	2	11%	5	31%	1	8%	1	20%	11	15%

Non-profit	1	4%	1	6%	2	13%	1	8%	-	-	5	7%

Corporations	7	30%	**8**	**44%**	-	-	-	-	-	-	15	20%

TOTAL	23	100%	18	100%	16	100%	13	100%	5	100%	75	100%

(Maximum in each column is highlighted. N = 75)

Table 4 shows the actual size of the participating hospitals. Although the number of beds varies in the different regions analyzed, the distribution in total is fairly equal for the categories defined.

**Table 4:** Number of beds per hospital.

	Slovakia		Switzerland		Austria		Bavaria		South Tyrol		TOTAL	
	
	n	%	n	%	n	%	n	%	n	%	n	%
0-299	**7**	**30%**	**13**	**72%**	5	31%	1	8%	1	20%	**27**	**36%**

300-599	6	26%	1	6%	**6**	**38%**	2	15%	**3**	**60%**	18	24%

600-899	4	17%	1	6%	2	13%	**6**	**46%**	1	20%	14	19%

> 900	6	26%	3	17%	3	19%	4	31%	0	0%	16	21%

TOTAL	23	100%	18	100%	16	100%	13	100%	5	100%	75	100%

The maximum in each column is highlighted.

The interviewees were also asked on their position they held within the hospital. The free-text answers were than categorized (see Table 5). The majority named their position "Head of the IT department" (43%, n = 32), "Chief Information Officer" (12%, n = 9) or "IT-Manager" (11%, n = 8). As several countries and organizations are involved, the different naming in the positions can primarily be accounted to a different understanding and culture and do not necessarily correspond to differences in responsibilities.

**Table 5:** Interviewees and their position within the organizations.

	Slovakia		Switzerland		Austria		Bavaria		South Tyrol		TOTAL	
	
	n	%	n	%	n	%	n	%	n	%	n	%
CEO/Managing Director	0	0%	3	17%	0	0%	2	15%	0	0	5	7%

CIO	0	0%	3	17%	3	19%	3	23%	0	0	9	12%

Head of IT department	**10**	**43%**	**7**	**39%**	**5**	**31%**	**7**	**54%**	**3**	**60%**	**32**	**43%**

IT Manager	3	13%	0	0%	3	19%	0	0%	2	40%	8	11%

Unspecified	6	26%	0	0%	0	0%	0	0%	0	0	6	8%

Others	4	17%	5	28%	5	31%	1	8%	0	0	15	20%

TOTAL	23	100%	18	100%	16	100%	13	100%	5	100%	75	100%

(Maximum in each column is highlighted. N = 75)

### Basic characteristics of the hospitals' IT organization

In general 95% (n = 71) of all hospitals have a central IT department. The lowest percentage of centralized IT departments could be observed in Switzerland with 89% (n = 16). An average of 44 hospitals (59%) have an IT strategy implemented, except for Austria where only 38% (n = 6) of all hospitals say to have an IT strategy.

Service provision is organized in Slovakian (70%, n = 16), Swiss (61%, n = 10) and Austrian (63%, n = 10) hospitals primarily in terms of a service-center concept whereas in Bavaria cost-centers and mixed approaches are equally common (each 42%, n = 5) and mixed approaches are predominant in South Tyrol (60%, n = 3). For details on the service provision please refer to Table 6.

**Table 6:** Organization of IT service provision.

	Slovakia		Switzerland		Austria		Bavaria		South Tyrol		TOTAL	
	
	n	%	n	%	n	%	n	%	n	%	n	%
Cost-Center	2	9%	5	28%	3	19%	1	8%	1	20%	12	16%

Service-Center	**16**	**70%**	**11**	**61%**	**10**	**63%**	**6**	**46%**	1	20%	**44**	**59%**

Mixed	1	4%	1	6%	2	13%	5	38%	**3**	**60%**	12	16%

Not familiar with the concepts	4	17%	1	6%	1	6%	1	8%	0	0%	7	9%

TOTAL	23	100%	18	100%	16	100%	13	100%	5	100%	75	100%

(Maximum in each column is highlighted. N = 75)

The majority of hospitals provide their services in-house (44%, n = 33) or with a mixed (48%, n = 36) approach (see Table 7).

**Table 7:** Organization of IT services - service provider.

	Slovakia		Switzerland		Austria		Bavaria		South Tyrol		TOTAL	
	
	n	%	n	%	n	%	n	%	n	%	n	%
In-House	9	39%	**9**	**50%**	**7**	**44%**	**7**	**54%**	1	20%	33	44%

Out-Sourced	3	13%	1	6%	1	6%	0	0%	0	0%	5	7%

Mixed	**11**	**48%**	8	44%	**7**	**44%**	6	46%	**4**	**80%**	**36**	**48%**

N.A.	0	0%	0	0%	1	6%	0	0%	0	0%	1	1%

TOTAL	23	100%	18	100%	16	100%	13	100%	5	100%	75	100%

The maximum in each column is highlighted.

From those 43 hospitals that answered the question, 61% spend less than 2% of their total budget annually for IT. The highest IT budgets can be observed in Switzerland and Austria (see Table 8 for details).

**Table 8:** IT budget in percent from the total hospital budget.

	Slovakia		Switzerland		Austria		Bavaria		South Tyrol		TOTAL	
	
	n	%	n	%	n	%	n	%	n	%	n	%
0,1% - 1%	**4**	**66%**	0	0%	3	27%	1	17%	**3**	**75%**	11	26%

1,1% - 2%	0	0%	**7**	**44%**	3	27%	**4**	**67%**	1	25%	**15**	**35%**

2,1% - 3%	1	17%	3	19%	**4**	**36%**	1	17%	0	0%	9	21%

3,1% - 4%	0	0%	5	31%	1	9%	0	0%	0	0%	6	14%

> 4%	1	17%	1	6%	0	0%	0	0%	0	0%	2	5%

TOTAL	6	100%	16	100%	11	100%	6	100%	4	100%	43	100%

(Maximum in each column is highlighted. N = 43)

Another question asked the hospitals about the availability of an IT service catalogue within their organization. Most of the hospitals stated not to have an IT service catalogue; only 7 hospitals in Switzerland, 5 hospitals in Bavaria and 3 hospitals in Austria stated to have one.

### Knowledge about IT Service management in general

55% (n = 41) of respondents claimed to be familiar with IT-Service Management methods. The highest percentage was found in Bavaria with 92% (n = 12), the lowest in Slovakia with 17% (n = 4). See Table 9 for details.

**Table 9:** "Are you familiar with the methods of IT-Service management?" (The maximum in each column is highlighted. N = 75)

	Slovakia		Switzerland		Austria		Bavaria		South Tyrol		TOTAL	
	
	n	%	n	%	n	%	n	%	n	%	n	%
YES	4	17%	**12**	**67%**	**10**	**63%**	**12**	**92%**	**3**	**60%**	**41**	**55%**

NO	**16**	**70%**	4	22%	3	19%	1	8%	2	40%	26	35%

N.A.	3	13%	2	11%	3	19%	0	0%	0	0%	8	11%

TOTAL	23	100%	18	100%	16	100%	13	100%	5	100%	75	100%

### ITIL^®^

In most of the countries, more than two thirds of the respondents claim to be familiar with ITIL^® ^(see Table 10). Only in Slovakia, 87% (n = 20) are not familiar with ITIL^®^.

**Table 10:** "Are you familiar with the term/concept ITIL^®^?" (The maximum in each column is highlighted. N = 75)

	Slovakia		Switzerland		Austria		Bavaria		South Tyrol		TOTAL	
	
	n	%	n	%	n	%	n	%	n	%	n	%
YES	3	13%	**14**	**78%**	**11**	**69%**	**11**	**85%**	**4**	**80%**	**43**	**57%**

NO	**20**	**87%**	4	22%	5	31%	2	15%	1	20%	32	43%

TOTAL	23	100%	18	100%	16	100%	13	100%	5	100%	75	100%

Additional, more detailed questions with regard to ITIL^® ^were only asked to interviewees who had previously stated to be familiar with ITIL^® ^(N = 43; this concerns tables 8, 11, 12, 13, 14).

**Table 11:** Institutions that have ITIL^® ^certified employees.

	Slovakia		Switzerland		Austria		Bavaria		South Tyrol		TOTAL	
	
	n	%	n	%	n	%	n	%	n	%	n	%
YES	0	0%	4	29%	1	9%	2	18%	0	0%	7	16%

NO	**3**	**100%**	**10**	**71%**	**10**	**91%**	**9**	**82%**	**4**	**100%**	**36**	**84%**

TOTAL	3	100%	14	100%	11	100%	11	100%	4	100%	43	100%

(Maximum in each column is highlighted. N = 43)

**Table 12:** "Do you plan to implement ITIL^® ^in the near future?" (The maximum in each column is highlighted. N = 43)

	Slovakia		Switzerland		Austria		Bavaria		South Tyrol		TOTAL	
	
	n	%	n	%	n	%	n	%	n	%	n	%
(Parts of) ITIL^® ^are already implemented	0	0%	3	21%	1	9%	1	9%	0	0%	5	12%

We plan to implement (parts of) ITIL^® ^in the next two years	1	33%	3	21%	1	9%	2	18%	1	25%	8	19%

We do not plan ITIL^® ^implementation in the next two years	**2**	**67%**	**7**	**50%**	**6**	**55%**	**4**	**36%**	**3**	**75%**	**22**	**51%**

N.A.	0	0%	1	7%	3	27%	4	36%	0	0%	8	19%

TOTAL	3	100%	14	100%	11	100%	11	100%	4	100%	43	100%

**Table 13:** "What are major reasons for you to not introduce ITIL^® ^so far?" (The maximum in each column is highlighted. Answers given for selection. Multiple answers possible. N = 43)

	Slovakia		Switzerland		Austria		Bavaria		South Tyrol		TOTAL	
	
	n	%	n	%	n	%	n	%	n	%	n	%
Missing budget	3	50%	0	0%	1	9%	1	9%	1	25%	6	13%

Different priorities	2	33%	**10**	**71%**	**7**	**64%**	**9**	**82%**	**3**	**75%**	**31**	**67%**

Missing motivation by management or staff	**4**	**67%**	0	0%	3	27%	1	9%	0	0%	8	17%

Other reasons	0	0%	2	14%	3	27%	2	18%	1	25%	8	17%

Overall number of respondents who gave at least one reason	6		12		11		11		4		46	

**Table 14:** "Do you think that ITIL^® ^will be used in healthcare as a domain-wide concept for IT service management?" (The maximum in each column is highlighted. N = 43)

	Slovakia		Switzerland		Austria		Bavaria		South Tyrol		TOTAL	
	
	n	%	n	%	n	%	n	%	n	%	n	%
YES	**2**	**67%**	4	29%	2	18%	**6**	**55%**	**2**	**50%**	16	37%

NO	0	0%	**8**	**57%**	**6**	**55%**	3	27%	1	25%	**18**	**42%**

N.A.	1	33%	4	29%	3	27%	2	18%	1	25%	11	26%

TOTAL	3	100%	14	100%	11	100%	11	100%	4	100%	43	100%

Out of the 43 institutions where the respondent felt familiar with ITIL^®^, only 7 (18%) already have ITIL^® ^certified employees in their organizations. Detailed figures are shown in Table 11. More than 70% of the organizations asked do also not plan to have their employees certified in the near future.

Hospitals were then asked on the expectations they have with regard to ITIL^® ^introduction. The majority of the hospitals named better service quality (61%, n = 28) and an increase in productivity (33%, n = 15). See Table 15 for details.

**Table 15:** "Which reasons lead to the introduction of ITIL^® ^in your hospital?" (The maximum in each column is highlighted. Answers given for selection. Multiple answers possible. N = 43)

	Slovakia		Switzerland		Austria		Bavaria		South Tyrol		TOTAL	
	
	n	%	n	%	n	%	n	%	n	%	n	%
Better IT service	**5**	**83%**	**6**	**43%**	**7**	**64%**	**7**	**64%**	**3**	**75%**	**28**	**61%**

IT cost reduction	2	33%	1	7%	0	0%	3	27%	0	0%	6	13%

Increase in productivity	2	33%	5	36%	2	18%	4	36%	2	50%	15	33%

Other benefits	0	0%	2	14%	3	27%	2	18%	0	0%	7	15%

Overall number of respondents who gave at least one benefit	6		14		11		11		4		46	

Hospitals were additionally asked if they have already implemented ITIL^® ^in their organizations, or if they are planning to implement ITIL^®^. In South Tyrol and Slovakia no hospital, in Austria and Bavaria only one hospital, and in Switzerland three hospitals have implemented at least parts of ITIL^®^. Eight hospitals (22%) plan an introduction in the next two years. The remaining 22 hospitals (51%) do not plan an introduction in the near future. All answers are summarized in Table 12.

These hospitals that had already implemented parts of ITIL^® ^(n = 5) were asked further details. In terms of the ITIL^® ^processes introduced, four of the five institutions - except one in Switzerland - have implemented *Incident *and *Problem Management*. Change Management is introduced by three hospitals in Switzerland. These three hospitals in Switzerland have also implemented further but differing ITIL^® ^processes. Being asked about the fulfillment of their expectations with regard to ITIL^® ^two hospitals in Switzerland were not satisfied.

When asked about the problems during the introduction of ITIL^®^, the organizations named poor motivation of employees, difficulties to select appropriate and beneficial ITIL^® ^processes, the burden of replacement of current processes, language difficulties and continuance of informal processes as reasons for problems.

The hospitals which have not yet implemented ITIL^® ^were asked for reasons. They predominantly answered with "Different priorities" (67%, n = 31). Budget constraints or missing motivation were also chosen as reasons but except for Slovakia of minor importance to the hospitals (see Table 13 for details). Other reasons were given by 17% (n = 8) including missing knowledge about ITIL, missing personnel or the use of a different philosophy. Similar reasons for failing or hindering the introduction of ITIL were also found by [27-29].

These results go together with the estimation of the interviewees about the potential use of ITIL^® ^within the healthcare sector (see Table 14). 37% (n = 16) of the interviewees claiming to be familiar with ITIL^® ^think that ITIL^® ^will be used as a domain-wide approach for IT service management, while 42% (n = 18) think that this will not be the case.

## Discussion

### Participating hospitals

The participating hospitals were a small, non-randomized sample of all hospitals in the given region or country. Hospitals were stratified according to geographic areas. The participating hospitals included a roughly equal percentage of university hospitals, specialized hospitals, general hospitals, and other types of hospitals, with an equally roughly equal distribution between smaller and larger hospitals. Overall, 75 hospitals participated, the majority of them in public ownership.

### How is IT organized in the participating hospitals?

The large majority of hospitals have central IT departments responsible for the provision of IT-related services. This should typically facilitate the adoption of ITIL^® ^as the implementation can be initiated by a single entity.

What is also of special importance to the adoption of ITIL^® ^is the presence of an IT strategy. As only 59% of all hospitals said to have a working IT strategy, this can be regarded as one barrier for the current introduction of ITIL^®^. The study indicates that in three (Slovakia, Switzerland and Austria) of the five countries, IT departments are structured predominantly according to a service-center concept, which would indicate that IT departments just provide supportive, non-marketable IT services. This is supported by the fact that in the large majority of hospitals, IT services are provided in house. What is indeed astonishing is the fact that only 15 hospitals (18%) said to have a service-catalogue although the majority of them have said to be organized according to a service-center or mixed approach involving the service-center idea. The annual IT-budget was below 2% for more than half of the houses, with lowest IT budgets in Slovakia and South Tyrol.

Overall, it seems that some organizational issues such as IT organization and IT budget are not yet sufficiently prepared in all hospitals to support the introduction of ITIL^®^.

### How advanced is the knowledge and attitude about IT service management and ITIL^® ^among IT managers in the different hospitals?

In most regions, two-thirds or more of the participating IT managers claim to be familiar with the concepts about IT service management, about quality assessment, and about ITIL^®^. Only in Slovakia, the large majority stated not to be familiar with any of those concepts. The respondents self-assessed their level of familiarity; we did not try to objectively confirm their level of knowledge.

When being asked about their expectations, the IT managers stated that they mostly expected better IT services, followed by an increase in productivity and IT cost reduction. Similar expectations with regard to the benefits of an ITIL introduction can also be found in [29].

Those respondents who felt familiar with ITIL^® ^were asked whether they expect that ITIL^® ^will be a healthcare-wide concept for IT service management in the future. Here, the opinions were mixed. Interestingly, in Switzerland with a relatively high ITIL^® ^experience, only one-third agreed here.

As only few hospitals state to have ITIL^®^-certified staff members, further promotion and training in this area seem to be needed, as good planning and preparation contributes to smooth ITIL^® ^implementation [4]. Nevertheless training can only be regarded as one step in the complex process of building awareness and acceptance for ITSM respectively ITIL^®^. This is also stressed by [27] as one of the central success factors.

### How advanced are the different hospitals with regard to ITIL^® ^implementation?

Only five hospitals said to have implemented at least parts of ITIL^®^, and eight hospitals stated to be planning this in the next two years, which represents less than one-third of all hospitals. Switzerland seems to be leading here, with three hospitals having introduced ITIL^®^, and three other hospitals planning to do so.

Two out of the 5 ITIL^® ^hospitals said that their expectations with regard to the benefits of ITIL^® ^were not met. Also in areas outside healthcare, disappointment with the outcomes of ITIL^® ^implementation can be found [30].

As reasons why ITIL^® ^has not yet been implemented in the majority of hospitals respondents said mostly "other priorities". Which priorities are meant here were not further investigated in this study. Research from other areas points to the fact that important success factors are, among others, senior management commitment, a change management strategy training and creating an ITIL^®^-friendly culture [30,31]. Our results found that only few staff members are ITIL^® ^certified; it thus seems that one precondition to promote ITIL^® ^is further training. Lacking senior management commitment was only mentioned to a larger extend by the Slovakian participants; in the other countries and regions, this seemed not to be a problem.

Overall, ITIL^® ^implementation in health care settings was found to be rather weak, and lower than in other areas outside healthcare. In our study, we found that two-thirds of hospitals do not consider ITIL^® ^implementation in the near future.

### Which differences between hospitals of different regions/countries can be observed?

When it comes to ITIL^®^, Switzerland and Bavaria seem to be ahead of the other countries. There, we observed the highest levels of knowledge, the highest number of implementations or plans of an implementation as well as the highest number of ITIL^® ^certified staff members. The next group from Austria and South Tyrol, they comparably show lower numbers in ITIL^® ^knowledge and implementation. However, in this group, still the majority of IT-managers feel familiar with IT service management and ITIL^®^, even though very few implementations or implementation plans are reported. Slovakia represents a country where the majority of IT managers do not feel familiar with IT service management and ITIL^®^, and where consequently no implementations are yet reported.

### Related studies

In the healthcare domain ITSM and in particular ITIL was not in the focus of many researchers up to now and only a handful of related research activities were found querying Pubmed (e.g. [32,33]).

One study investigating the status of IT governance in 23 Swiss hospitals could be found [34]. The study has identified ITIL as the most prominent approach to ITSM. It is reported that 47% of the hospitals observed do not have an IT governance framework implemented. The most popular standard - if used - was ITIL.

To find more scientific work on ITSM and ITIL, one must look beyond the horizon of the healthcare domain. Also in other domains scientific research on the adoption of ITSM and the IT managers' perspectives on service management is limited [35]. Many studies just explore how and which ITSM approaches have been implemented.

Beyond the sheer descriptive approaches, some research groups were investigating the reasons why and how organizations are implementing ITIL in more detail and started to unveil the factors for successful or failed ITIL implementation (e.g. [36]).

A study from the ICT domain [37] found out that 76% of its participants regarded the adoption of a governance framework as critical for the success of a company's future.

Another study conducted for six German companies from different domains identified a better client/service orientation, the increased quality of IT services, an increase in efficiency and transparency as the most important factors for introducing ITIL [29]. These results were also observed in the current study.

In addition a great number of studies could be found focusing on important aspects of an ITIL implementation (e.g. [38,39]) which, certainly, should also be investigated in organizations operating in the healthcare domain.

### Study limitations

Several limitations have to be addressed with regard to the current study. Interviews were conducted in five different countries/regions. The selection comprised Austria, which is situated in the middle of Europe, as well as neighboring countries and regions. It was not possible to include all hospitals in a certain country/region - except for South Tyrol - therefore a sample of all hospitals had to be drawn. Thus, this study included between 10% (in Austria) and 100% (in South Tyrol) of all hospitals in the analyzed countries and regions. We tried to guarantee an even distribution of different types of hospitals and of geographic locations, but the sample is not a completely representative random sample. The return rate was satisfactory with 47.5% after sending a maximum of two reminders respectively calling the interviewees twice depending on the first type of contact. However, the non-participation of more than half of the selected hospitals may have lead to a drop-out bias.

The IT manager of each respective hospital was contacted and interviewed. This person was identified by the hospital itself. We did not further verify whether the nominated person was the correct person and qualified to provide the answers needed.

Interviews were conducted by different interviewers; one interviewer for each country/region. Even after designing an interview guideline and intense training, it may be possible that the interviews were not executed in an entirely identical way. Finally, some of the questions we posed were open; these were analyzed by structured qualitative methods. Some of the interviews were conducted personally or by telephone; no tape-recorder was used here. This made verification of documented answers difficult.

## Conclusion

The results collected through this study indicate that the idea that IT is not primarily a technology provider but rather a service provider is still not widely recognized in hospitals of the study countries and regions. This is of special importance as IT plays an increasingly important role in patient treatment (e.g. cross-institutional exchange of health related data, clinical decision support etc.) and therefore can both add to and reduce patient safety respectively quality of treatment. So hospitals are advised to carefully consider the implementation of IT governance approaches in order to cope with this uprising challenge. Since frameworks such as ITIL^® ^are built on best practices they can provide the methodological background and the practical guidance for the introduction of ITSM.

The study gives a first insight in the adoption of the service concept and the penetration of ITIL^® ^in hospitals. Overall, results on the implementation of IT service management and ITIL^® ^in health care are rare, and it seems necessary to conduct further research in this direction; among others, it seems still unclear whether the success factors for an ITIL^® ^implementation that were found in businesses outside health care can consistently be transferred to the health care context. The results of the current study are therefore targeted towards the provision of data to allow further and in-depth studies.

## Competing interests

The authors declare that they have no competing interests.

## Author information

EA, RB and AH have developed the design of the study. AH has written the first draft of the article and has edited the final version by integrating the various comments and suggestions. EA, RB, WH have substantially contributed to the article by revising the first draft and providing various comments. All authors have approved the final version of the manuscript.

## Pre-publication history

The pre-publication history for this paper can be accessed here:

http://www.biomedcentral.com/1472-6947/11/76/prepub
